# FOLFIRINOX: The Best Adjuvant Treatment for Ampullary Adenocarcinoma? A Multicenter Study by the Turkish Oncology Group (TOG)

**DOI:** 10.3390/cancers17172730

**Published:** 2025-08-22

**Authors:** Ali Kalem, Tulay Kus, Taha Koray Sahin, Omer Dizdar, Safa Can Efil, Mehmet Ali Nahit Sendur, Talat Aykut, Murat Araz, Hatice Bolek, Yuksel Urun, Nadiye Sever, Ibrahim Vedat Bayoglu, Eyyup Cavdar, Muhammed Fatih Sagıroglu, Tugce Kubra Gunes, Melike Ozcelik, Nadide Demirel, Bulent Yıldız, Berkan Karabuga, Ulku Yalcıntas Arslan, Savas Gokcek, Ilkay Tugba Unek, Seray Saray, Ferit Aslan, Omer Acar, Atike Pınar Erdogan, Mustafa Seyyar, Gokmen Aktas, Suayib Yalcın

**Affiliations:** 1 Department of Medical Oncology, School of Medicine, Gaziantep University, Gaziantep 27310, Turkey; tulaykus@gantep.edu.tr; 2 Department of Medical Oncology, School of Medicine, Hacettepe University, Ankara 06100, Turkey; takorsah@gmail.com (T.K.S.); dromerdizdar@gmail.com (O.D.); suayibyalcin@gmail.com (S.Y.); 3 Department of Medical Oncology, School of Medicine, Ankara YıldırımBeyazıt University, Ankara 06110, Turkey; safacan.efil@saglik.gov.tr (S.C.E.); mansendur@aybu.edu.tr (M.A.N.S.); 4 Department of Medical Oncology, School of Medicine, Necmettin Erbakan University, Konya 42090, Turkey; talat_aykut@hotmail.com (T.A.); zaratarum@yahoo.com (M.A.); 5 Department of Medical Oncology, School of Medicine, Ankara University, Ankara 06230, Turkey; hati.kocc@gmail.com (H.B.); yukselurun@gmail.com (Y.U.); 6 Department of Medical Oncology, School of Medicine, Marmara University, İstanbul 34854, Turkey; nadiye.sever@marmara.edu.tr (N.S.); dr.vebay@gmail.com (I.V.B.); 7 Department of Medical Oncology, Adıyaman Education and Research Hospital, Adıyaman 02100, Turkey; ecavdar@nku.edu.tr; 8 Department of Medical Oncology, Gaziantep City Hospital, Gaziantep 27470, Turkey; dr.mfsagiroglu@gmail.com (M.F.S.); mustafa.seyyar@saglik.gov.tr (M.S.); 9 Department of Medical Oncology, Ümraniye Education and Research Hospital, İstanbul 34764, Turkey; drtugcekubragunes@gmail.com (T.K.G.); drmelike.ozcelik@gmail.com (M.O.); 10 Department of Medical Oncology, School of Medicine, Eskisehir Osmangazi University, Eskisehir 26040, Turkey; nadide.demirel@saglik.gov.tr (N.D.); bulentyildiz@ogu.edu.tr (B.Y.); 11 Department of Medical Oncology, Dr. AbdurrahmanYurtaslan Oncology Education and Research Hospital, Ankara 06200, Turkey; drbkarabuga@gmail.com (B.K.); ulkuarslan63@gmail.com (U.A.Y.); 12 Department of Medical Oncology, School of Medicine, Dokuz Eylul University, Izmir 35220, Turkey; savas.gokcek@saglik.gov.tr (S.G.); ilkaytugbaunek@gmail.com (I.T.U.); 13 Department of Medical Oncology, Ataturk Education and Research Hospital, Balıkesir 10100, Turkey; seraysaray@saglik.gov.tr; 14 Department of Medical Oncology, Medical Park Ankara Batıkent Hospital, Ankara 06680, Turkey; feritferhat21@gmail.com; 15 Department of Medical Oncology, School of Medicine, Manisa Celal Bayar University, Manisa 45030, Turkey; dracaromer@gmail.com (O.A.); atike.erdogan@cbu.edu.tr (A.P.E.); 16 Gaziantep Medical Point Private Hospital, Gaziantep 27584, Turkey; gokmen.aktas@mph.com.tr

**Keywords:** ampullary adenocarcinoma, FOLFIRINOX, adjuvant, radiotherapy, gemcitabine

## Abstract

Background: Ampullary adenocarcinoma is a rare cancer for which there are no standard adjuvant treatment recommendations due to the lack of randomized clinical trials. The primary aim of this analysis is to investigate the efficacy of adjuvant FOLFIRINOX treatment in patients with resected ampullary adenocarcinoma. Materials and Methods: This multicenter retrospective cohort study was conducted at 15 institutions in Turkey between August 2007 and January 2024, involving 211 patients with resected, non-metastatic ampullary adenocarcinoma receiving adjuvant chemotherapy with various chemotherapy regimens with or without chemoradiation. Clinicopathological and treatment-related parameters were recorded. Disease-free survival (DFS) and overall survival (OS) were analyzed by using Kaplan–Meier estimates. Cox proportional hazards regression was used to identify covariates associated with OS. Results: The median follow-up time was 52 months, and 116 patients (55.2%) were alive at the time of the analysis. The median age was 61 years (32–82). mFOLFIRINOX was administered to 16.6% of the patients (*n* = 35). The 3-year DFS rate was 79.41% in the FOLFIRINOX-treated arm and 53.9% in the other treatment arm (*p* = 0.034 for mDFS). The median OS was non-reached in patients receiving mFOLFIRINOX treatment, while it was 51 months in patients receiving other treatments (*p* = 0.071). While no statistically significant results were reached, a trend toward statistically significant survival times was observed in the FOLFIRINOX arm. After adjustment for other prognostic parameters, mFOLFIRINOX remained an independent statistically significant parameter for better OS (HR; 95% CI: 3.24; 1.02–10.9; *p* = 0.046). Conclusions: FOLFIRINOX treatment has shown efficacy in the adjuvant treatment of ampullary cancer, independent of histological subtype. The findings should be validated in large prospective trials.

## Simple Summary

Ampullary adenocarcinoma is a rare cancer with no universally accepted adjuvant treatment. Our multi-center retrospective study evaluated the role of modified FOLFIRINOX (mFOLFIRINOX) compared to other adjuvant regimens. We found that mFOLFIRINOX was associated with significantly improved disease-free survival and a favorable trend toward better overall survival, independent of histological subtype. While the regimen has a higher toxicity profile, it may be considered for fit, younger patients. This is the largest dataset on adjuvant FOLFIRINOX in ampullary adenocarcinoma and can help guide future clinical trials.

## 1. Introduction

Ampullary cancers are defined as tumors originating from the ampulla of Vater, which comprises three anatomical components: the ampulla, the intraduodenal portion of the bile duct, and the intraduodenal portion of the pancreatic duct [1]. Although they are a single type of tumor, ampullary cancers can have different clinical courses. Kimura W et al. first proposed that ampullary tumors exhibit different characteristics and prognoses based on the epithelial cloaca from which they originate. Accordingly, ampullary tumors are histologically classified into two types: (1) an intestinal type that resembles tubular adenocarcinoma of the stomach or colon and (2) a pancreaticobiliary type characterized by papillary projections with scant fibrous cores. The age distributions of both types are similar; however, the prognosis of the intestinal type is much better than that of the pancreaticobiliary type [2,3]. Additionally, lymph node metastasis and a strong infiltrative tendency are more common in the pancreaticobiliary subtype, while the intestinal type tends to have little or no invasion into the surrounding interstitium [2,3]. This histological heterogeneity may be responsible for the differing clinical outcomes and treatment responses [4,5,6,7].

In addition to their histological heterogeneity, ampullary cancers are very rare tumors, accounting for only 0.2% of gastrointestinal malignancies [8]. Consequently, the results of clinical trials regarding ampullary adenocarcinoma are not definitive due to their rarity and are often analyzed as part of biliary tract cancers. There is no consensus on the optimal adjuvant treatment for patients with ampullary cancer. Recommendations for the management of ampullary carcinoma have only recently been included in the National Comprehensive Cancer Network (NCCN) guidelines as a separate category [9]. Given the more favorable prognosis of ampullary cancer compared to biliary tract cancers and the lack of strong data from randomized trials proving a survival advantage, the level of evidence for adjuvant chemoradiotherapy or specific chemotherapy regimens is weak [9,10].

In our study, we aimed to analyze the effectiveness of mFOLFIRINOX chemotherapy, which has been shown to be effective in both pancreatic cancer and bowel cancer, in the adjuvant treatment of patients with ampullary cancer who underwent curative intent surgery. This analysis is based on histological subtype and is compared with other systemic treatments [11].

## 2. Materials and Methods

### 2.1. Patients and Study Design

This multicenter, retrospective observational study included patients from 15 centers across various regions of Turkey between August 2007 and January 2024. Patients aged at least 18 years who had received a histological and clinical diagnosis of ampullary adenocarcinoma and underwent curative intent Whipple procedures were screened. Patients who underwent R0 or R1 resection and received adjuvant chemotherapy or chemoradiotherapy were included.

Patients were followed up using computed tomography, routine blood tests, and tumor marker assays every 3–4 months for the first 2 years, then every 6 months for 5 years, and annually thereafter. Additionally, we meticulously examined the recurrence patterns (local or distant) among patients who experienced recurrence and documented the treatments they received during these recurrence episodes.

The study was conducted in accordance with the principles outlined in the Declaration of Helsinki and received approval from the ethics committees of the participating centers. The Ethics Committee of Gaziantep University Faculty of Medicine approved the study protocol, which included patients’ clinical data, informed consent, and the multicenter design (Approval No. 2024/43). In addition, corresponding clinical information (e.g., age and gender) and pathological characteristics were collected from the hospital information management system. The datasets used and/or analyzed during the current study are available from the corresponding author upon reasonable request.

### 2.2. Patients and Disease Features

Baseline characteristics, treatment regimens, and treatment and recurrence dates were retrospectively examined from patient records and electronic systems. The following parameters were recorded: patient age, gender, comorbidities, alcohol use, smoking status, the Eastern Cooperative Oncology Group (ECOG) performance status (0–2), and disease pathological T stage (T1-4) and N stage (0–2) using the American Joint Committee on Cancer staging system (AJCC, 8th edition of the TNM classification). Additionally, histopathological subtype (pancreaticobiliary type or intestinal type), presence of lymphovascular invasion (LVI) and perineural invasion (PNI), resection margin (R0 or R1), presence of pancreatic invasion and biliary obstruction, lymph node ratio (LNR; positive lymph node/total harvested lymph nodes), and total harvested lymph nodes (THLN; <16 vs. ≥16) were recorded.

Laboratory parameters, including hemoglobin, neutrophil, lymphocyte, and platelet levels, detailed liver function tests, and tumor markers (CEA ng/mL, CA 19-9 U/mL) were also noted.

### 2.3. Adjuvant Treatments

Other treatments administered in addition to mFOLFIRINOX (oxaliplatin plus irinotecan with leucovorin and short-term infusional5-fluorouracil [FU]) chemotherapy included gemcitabine in combination with FU-based therapy or cisplatin; gemcitabine or FU monotherapy; chemoradiotherapy (with gemcitabine or FU/capecitabine); and FU-based doublets (XELOX, FOLFOX). Additionally, radiotherapy was administered to some patients outside of chemoradiotherapy.

All investigators used the Common Terminology Criteria for Adverse Events “CTCAE” version 5.0 to assess toxicity.

### 2.4. Statistical Analysis

The normal distribution of numerical variables was assessed using the Shapiro–Wilk test. Relationships between categorical variables were examined using the χ^2^ test and Fisher’s Exact Test, with categorical variables presented as counts (ns) and percentages (%s). Continuous variables were reported as mean ± standard deviation (SD). Disease-free survival (DFS) and overall survival (OS) were estimated using the Kaplan–Meier method, and the log-rank test was used to compare the effects of clinicopathological and laboratory parameters on survival in the univariate analysis. Univariate Cox regression was employed to estimate hazard ratios (HRs) with 95% confidence intervals (CIs) for OS and DFS. All potential predictive factors with a *p*-value <0.10 in the univariate analyses were included in the multivariate analysis. The Cox proportional hazards model was used for multivariate analysis to determine independent significant factors. Cox regression curve of overall survival of patients with intestinal-type ampullary carcinoma and pancreaticobiliary/mix type ampullary carcinoma was performed after adjustment for age, comorbidities, performance status, stage, lymphovascular and perineural invasion status, resection margin, pancreatic invasion, and CA 19-9.Cox regression curve was performed for DFS after adjustment for performance status, stage, lymphovascular and perineural invasion status, resection margin, pancreatic invasion, and CA 19-9. Next, Cox regression curve of the OS of patients was performed after adjustment for age, comorbidities, performance status, stage, lymphovascular and perineural invasion status, resection margin, pancreatic invasion, and CA 19-9.A *p*-value of ≤0.05 was considered statistically significant, and statistical analyses were conducted using the SPSS Statistics version 22.0 (IBM Corp., Armonk, NY, USA).

The primary endpoint was to assess the efficacy of FOLFIRINOX on DFS by comparing it with other treatment options. DFS was defined as the time from the start of adjuvant therapy to the occurrence of the first recurrence (local, regional, or distant metastasis), death from any cause, or the date of the last follow-up. OS was defined as the period from the initiation of adjuvant treatment to the date of death or the last follow-up.

## 3. Results

### 3.1. Patient Demographics and Tumor Characteristics

A total of 211 patients with ampullary adenocarcinoma who underwent curative intent surgery and received adjuvant treatment were included in this retrospective study. Patients with R2 resection, inadequate follow-up (<30 days), or those who did not receive adjuvant systemic chemotherapy were excluded. The median age was 61 years (range, 32–82 years); 123 patients (58.3%) were men, and the remaining were women. Seventy-two patients (34.1%) were 65 years or older, and 109 patients (51.7%) had comorbidities (such as coronary artery disease, diabetes, hypertension, or inflammatory diseases). The ECOG performance score was 0 or 1 for 93.9% of the patients, and the remaining patients had a score of 2 (Table 1). The most common histological subtype was pancreatobiliary or mixed intestinal and pancreatobiliary (*n* = 141; 76.2%), followed by intestinal (*n* = 44; 23.8%). Most patients (*n* = 136; 63.4%) had stage III disease. All other potentially prognostic parameters are shown in Table 1.

In terms of the clinicopathological characteristics of the patients, there was generally no statistically significant difference between the group receiving mFOLFIRINOX and the group receiving other treatments. However, it was observed that the mFOLFIRINOX treatment was less commonly recommended for patients over 65 years of age. Additionally, the rate of radiotherapy administered in the mFOLFIRINOX group was statistically significantly lower (Table 1). Nevertheless, neither age nor radiotherapy treatment was a statistically significant parameter for DFS (*p* > 0.05) (Table 2).

### 3.2. Effect of Clinicopathologic, Inflammatory, and Treatment-Related Parameters on Disease-Free Survival

The median DFS was 30 (24.3–35.7) months. Of the 211 patients, 114 experienced a recurrence (54.3%), and the DFS data for one patient could not be accessed; only the overall survival data was available for that patient.

The most frequently administered regimen was gemcitabine in combination with FU-based or cisplatin (N = 62; 29.4%), followed by gemcitabine/FU monotherapy (N = 43, 20.4%), chemoradiotherapy (with gemcitabine or FU/capecitabine, N = 40; 19%), FU-based doublet (XELOX, FOLOX, *n* = 31; 14.7%), and mFOLFIRINOX (*n* = 35; 16.6%).

**Figure 1:**
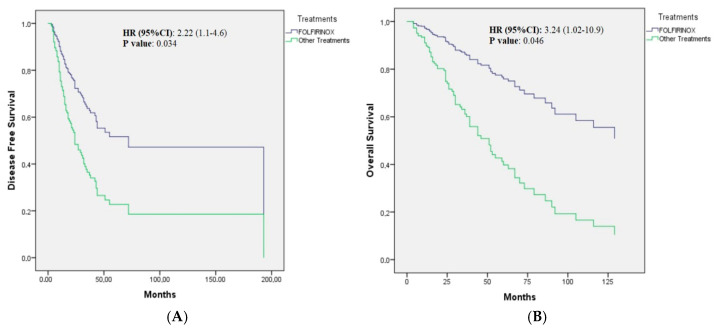
(**A**): Cox regression curve of disease-free survival in the adjuvant treatments of patients with ampullary adenocarcinoma after adjustment for Eastern Cooperative Oncology Group performance status, stage, lymphovascular and perineural invasion status, resection margin, pancreatic invasion, and CA 19-9. (**B**): Cox regression curve of overall survival in the adjuvant treatments of patients with ampullary adenocarcinoma after adjustment for age, comorbidities, Eastern Cooperative Oncology Group performance status, stage, lymphovascular and perineural invasion status, resection margin, pancreatic invasion, and CA 19-9.

The median DFS was non-reached (NR) (NR; NR-NR) in patients treated with mFOLFIRINOX, while it was 27 (20.1–33.3) months in patients receiving other treatment modalities (*p* = 0.034). (The 3-year DFS rate was 79.41% in the FOLFIRINOX-treated arm and 53.9% in the other treatment arm.) As shown in Table 2, while age, gender, presence of comorbidities, BMI, smoking and alcohol use, histopathological subtype, and radiotherapy use were not effective parameters for DFS, better ECOG performance status, lower T and N stages, absence of pancreatic invasion and biliary obstruction, negative surgical margins, absence of lymphovascular and perineural invasion, low CA 19-9 level, and use of FOLFIRINOX adjuvant therapy were associated with better DFS in the univariate analysis (Table 2). The optimal total harvested lymph nodes value (≥16) was not predictive for DFS; however, the lymph node ratio (LNR; positive lymph nodes/total harvested lymph nodes) was a predictive parameter for DFS. Since LNR was highly correlated with lymph node stage, it was not included in the multivariate analysis. Additionally, there was a high correlation between the presence of biliary obstruction and the CA 19-9 level; therefore, the CA 19-9 level was chosen for the model. In the multivariable analysis, only negative surgical margins, absence of LVI, and mFOLFIRINOX treatment were associated with better DFS (HR 95%CI: 2.22:1.1–4.6; *p* = 0.034) (Table 2; Figure 1A).

In patients who received radiotherapy, the rate of local recurrence was statistically significantly lower (40% vs. 20%; *p* = 0.034).

### 3.3. Effect of Clinicopathologic, Inflammatory, and Treatment-Related Parameters on Overall Survival

The median follow-up time was 52 months. At the end of the follow-up, 116 patients (55.2%) were alive. The median OS was NR in patients receiving mFOLFIRINOX treatment, while it was 51 months in patients receiving other treatments (*p* = 0.071). (The 5-year overall survival rate was 88.2% in the FOLFIRINOX treatment arm and 55.7% in the other treatment arm.) While no statistically significant result was reached, a trend toward statistically significant survival times was observed in the FOLFIRINOX arm. Since the *p*-value was below 0.10, it was included in the multivariate analysis. Accordingly, as shown in Table 3, while BMI, smoking and alcohol use, histopathological subtype, LNR, THLN, biliary obstruction, and radiotherapy use were not effective parameters for OS, younger age, absence of comorbidities, better ECOG performance status, lower T and N stages, absence of pancreatic invasion, negative surgical margins, absence of lymphovascular and perineural invasion, low CA 19-9 level, and use of mFOLFIRINOX were associated with better OS in the univariate analysis. In the multivariate analysis, mFOLFIRINOX treatment was an independent statistically significant parameter for better OS (HR; 95% CI: 3.24; 1.02–10.9; *p* = 0.046) (Table 3; Figure 1B).

**Figure 2:**
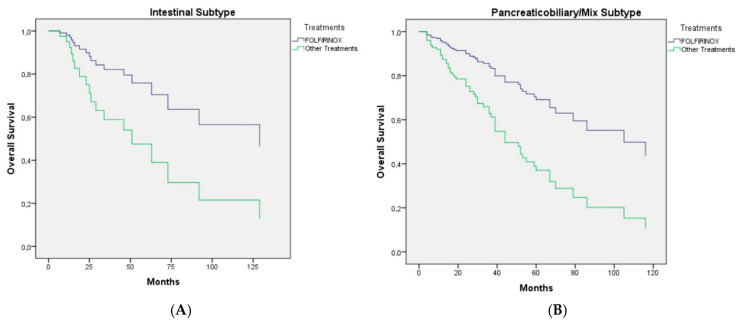
(**A**): Cox regression curve of overall survival of patients with intestinal-type ampullary carcinoma. (**B**): Cox regression curve of overall survival of patients with pancreaticobiliary/mix type ampullary carcinoma after adjustment for age, comorbidities, Eastern Cooperative Oncology Group performance status, stage, lymphovascular and perineural invasion status, resection margin, pancreatic invasion, and CA 19-9.

As shown in Figure 2, the clinical efficacy of FOLFIRINOX was observed in both histopathological subtypes (log-rank *p*-value: 0.040).

### 3.4. Toxicity

In the FOLFIRINOX group, dose reduction was implemented in 41.2% of patients (*n* = 14), and treatment was temporarily interrupted due to toxicity in 31.4% of patients (*n* = 11). In the other treatment group, dose reduction occurred in 20.5% of patients (*n* = 36) (*p* = 0.009), and treatment was interrupted in 17% of patients (*n* = 30) (*p* = 0.50).

In the FOLFIRINOX group, chemotherapy was discontinued due to toxicity in 5 patients (14.3%), while in the other treatment group, chemotherapy was discontinued in 12 patients (6.8%) (*p* = 0.138).

In the FOLFIRINOX group, grade 3–4 neutropenia occurred in 7 patients (20%) (Grade 1–2: *n* = 11; 31.4%), febrile neutropenia in 5 patients (14.7%), grade 3–4 anemia in 2 patients (5.9%) (Grade 1–2: *n* =19; 55.9%), and grade 3–4 thrombocytopenia in 2 patients (5.8%) (Grade 1–2: *n* = 10; 29.4%). In the other treatment group, grade 3–4 neutropenia was observed in 25 patients (14.2%) (Grade 1–2: *n* = 65; 36.9%), febrile neutropenia in 6 patients (3.5%), grade 3–4 anemia in 3 patients (1.8%) (Grade 1–2: N = 92; 55.5%), and grade 3–4 thrombocytopenia in 4 patients (2.4%) (Grade 1–2: *n* = 51; 31.3%) (*p* = 0.013; *p* = 0.008; *p* = 0.164; *p* = 0.234, respectively).

Grade 3–4 fatigue was higher in the FOLFIRINOX group compared to the other treatment group (10.3% vs. 2.6% for grade 3–4; 75.8% vs. 62.1% for grade 1–2, respectively; *p* = 0.043). However, appetite loss was similar between the groups (7.1% vs. 2.0% for grade 3–4; 60.7% vs. 54.3% for grade 1–2, respectively; *p* = 0.30), and grade 1–2 diarrhea was more common in the FOLFIRINOX group (32.2% vs. 16.2%, respectively; *p* = 0.034; no grade ≥ 3). Stomatitis (grade 1–2: 25.9% vs. 20.2%; *p* = 0.69; no grade ≥3) and liver function test abnormalities were similar between the groups (0.0% vs. 1.3% for grade 3–4; 34.3% vs. 20.9%, respectively; *p* = 0.37; no grade ≥ 3) (Table 4).

## 4. Discussion

Our study is the largest database analysis to date examining the effectiveness of FOLFIRINOX in the adjuvant treatment of ampullary cancers. FOLFIRINOX has revolutionized survival times in the adjuvant and metastatic stages of pancreatic cancer [12,13] and has also shown survival benefits, particularly in certain subgroups of metastatic intestinal cancers [14]. Although there are no randomized controlled trials specifically evaluating FOLFIRINOX for the adjuvant treatment of ampullary cancers, it is now recommended by the current National Comprehensive Cancer Network (NCCN) guidelines for the adjuvant treatment of the pancreaticobiliary subtype of ampullary tumors [9]. Our study demonstrates that FOLFIRINOX provides superior disease-free survival (DFS) and a favorable trend in overall survival (OS) benefits in the adjuvant treatment of ampullary cancer compared to other adjuvant chemotherapy or chemoradiotherapy modalities. When other prognostic parameters were included in the multivariate analysis, the overall survival benefit of FOLFIRINOX treatments reached statistical significance [9,10,11,15,16].

In our study, well-established predictive parameters for recurrence, such as ECOG performance status, stage, positive surgical margins, lymphovascular invasion, perineural invasion, pancreatic invasion, development of jaundice, and elevated CA 19-9 levels, were confirmed as risk factors for recurrence. In the multivariate analysis, positive surgical margins and FOLFIRINOX chemotherapy emerged as independent predictive parameters (Table 2). The characteristics related to patients and tumors receiving FOLFIRINOX were similar to those receiving other treatments, as shown in Table 1. However, it was observed that FOLFIRINOX was less frequently administered to patients over 65 years of age, although age was not found to be a predictive factor for DFS. Patient age, gender, habits, and comorbidities were not predictive of recurrence. While the dissection of at least 16 lymph nodes is recommended [17], our study did not find a predictive parameter for recurrence based on the number of lymph nodes dissected, as both patients with ≥16 and <16 lymph nodes were compared. Although an increased positive lymph node ratio (NLR) has been associated with reduced survival, our study found that NLR, while related to early recurrence and poor survival, was less effective than nodal stage, which was therefore included in the model. Elevated CA 19-9 levels were found to be prognostic for both DFS and OS in the univariate analysis, consistent with existing literature [18].

Radiation therapy (RT) can be used in localized ampullary cancer, sometimes in combination with chemotherapy, as suggested by NCCN guidelines; however, there is no high-level evidence to support its use [19]. The Surveillance, Epidemiology, and End Results (SEER) database was used to assess the benefit of RT in patients with ampullary cancer. According to the propensity score matching analysis, adjuvant radiotherapy did not improve either OS (*p* = 0.119) or disease-specific survival (*p* = 0.188) [20]. Consistent with the literature, our study found that adding RT did not have a positive impact on DFS or OS. However, it was observed that the local recurrence rate was statistically significantly lower in patients who received RT compared to those who did not.

The European Study Group for Pancreatic Cancer (ESPAC)-3 trial was the first phase 3 randomized controlled trial to compare the survival benefits of adjuvant chemotherapy versus observation following resection for patients with periampullary carcinoma (including ampullary, intrapancreatic bile duct, nondescript, or periampullary duodenal cancer) and analyze the efficacy of fluorouracil plus folinic acid chemotherapy and gemcitabine alone [21]. Of the 428 patients included in the primary analysis, 297 had ampullary cancer, 96 had bile duct cancer, and 35 had other types of cancer. Among the 105 patients with ampullary cancer in the observation group, the median survival was 40.6 months (95% CI, 30.6–61.4 months); among 100 patients in the fluorouracil plus folinic acid group, it was 57.8 months (95% CI, 32.8–84.0 months); and among 92 patients in the gemcitabine group, it was 70.8 months (95% CI, 45.3–not reached months). There were no statistically significant differences in overall survival between patients with the pancreatobiliary subtype and those with the intestinal subtype of ampullary tumors. Multivariate analysis, adjusting for prognostic variables, demonstrated a statistically significant survival benefit with chemotherapy, specifically for gemcitabine compared to observation [19]. In our study, although the DFS and OS of the intestinal subtype were observed to be longer compared to the pancreatobiliary/mixed subtype, this difference was not statistically significant. FOLFIRINOX treatment was shown to be superior to other treatments in both subtypes (Figure 2). Our study also found that gemcitabine doublet or 5-FU doublet therapies did not show superiority over monotherapy in terms of DFS (Table 2) [9,15].

With the exception of the ESPAC-3 study, no treatment recommendations in the NCCN guidelines are based on phase II or phase III studies specifically designed for ampullary tumors. In the ABC-2 study, which included ampullary tumors among patients with metastatic biliary tract cancers, the survival advantage of adding cisplatin to gemcitabine was demonstrated, and it was observed that ampullary tumors also benefited from this combination treatment [22]. Another recommended combination therapy, XELOX, was shown to be an effective treatment in a phase II study that included metastatic small bowel cancers and ampullary cancers. Consequently, gemcitabine doublet and 5-FU doublet treatments, which were effective in the metastatic stage according to subgroup analyses, have been recommended as adjuvant treatments by guidelines [23]. Similarly, XELOX has been included in the adjuvant treatment of intestinal-type ampullary cancer based on its efficacy in the adjuvant treatment of colon cancer [24,25]. In our study, the FU-doublet chemotherapy regimen did not show superiority over 5-FU monotherapy; however, FOLFIRINOX treatment provided a better DFS advantage, including in the intestinal subtype.

Another combination therapy, gemcitabine and capecitabine, demonstrated effectiveness in the ESPAC-4 study, where it was compared with gemcitabine alone. This combination, which has been shown to be effective in the adjuvant treatment of pancreatic cancer, has become one of the recommended combinations for the adjuvant treatment of ampullary cancers based on this study [26]. In our study, this combination was evaluated within the gemcitabine-based chemotherapy group and showed a negative DFS outcome compared to FOLFIRINOX [10].

Although there are no phase studies in the literature, there are extensive series of adjuvant treatment studies for ampullary cancers. In a database study by Ecker BL. et al., similar to our study, it was observed that neither chemotherapy nor radiotherapy contributed to overall survival in patient populations receiving 5-FU monotherapy or combinations with oxaliplatin or irinotecan, as well as gemcitabine monotherapy and gemcitabine-based doublet chemotherapy [15]. A similar meta-analysis also yielded comparable results [27]. Given the superiority of FOLFIRINOX over other treatments in our study, it is suggested that the negative outcomes in these studies may be related to the fact that patients did not receive optimal adjuvant systemic chemotherapy [11].

In our study, higher rates of neutropenia and febrile neutropenia were observed in the mFOLFIRINOX group, with a greater frequency of dose reductions and treatment discontinuations. Additionally, the administration of mFOLFIRINOX was notably lower in patients aged 65 and older compared to other treatment groups. In this context, it is advisable to use mFOLFIRINOX primarily in fit patients younger than 65, to initiate treatment with dose reductions, and to routinely use growth factors to manage side effects.

## 5. Limitations

The primary limitations of this study are its retrospective nature and the relatively small number of patients in the mFOLFIRINOX group compared to the control group. Additionally, the follow-up duration for the mFOLFIRINOX group did not reach the median follow-up time, and there was data loss due to the retrospective nature of the study. One of the major limitations of the study is that the hazard ratio for OS (HR = 3.24) showed a wide confidence interval (1.02–10.9), indicating considerable variance.

## 6. Conclusions

This multi-institutional study represents the largest series of surgically resected ampullary adenocarcinoma patients treated with adjuvant chemotherapy, including mFOLFIRINOX, with detailed histopathological data. Therefore, we have reported significantly important parameters regarding the overall management of patients with ampullary adenocarcinoma. FOLFIRINOX provides better DFS and a favorable trend in OS benefits in the adjuvant treatment of ampullary cancer compared to other adjuvant chemotherapy or chemoradiotherapy modalities. When other prognostic parameters were included in the multivariate analysis, the OS benefit of FOLFIRINOX reached statistical significance, independent of histological subtype.

Given the high toxicity rate of FOLFIRINOX, its use should be carefully considered based on patients’ performance status and comorbidities. It should also be noted that FOLFIRINOX treatment was not preferred in the elderly population in our database, and, therefore, the data is insufficient for this population. Although our study provides the highest level of patient data in the literature, confirmation of our data through prospective randomized trials is needed to recommend FOLFIRINOX treatment with a high level of evidence; therefore, our data can shed light on future planned studies [28].

## Figures and Tables

**Table 1:** Baseline characteristics of patients with resected ampullary adenocarcinoma.

	mFOLFIRINOX N (%)	The Other Treatments N (%)	*p* Value
Age, years			
(median; range)			
<65	33 (9.4.3)	106 (60.2)	<0.001
≥65	2 (5.7)	70 (39.8)	
Gender			
Female	19 (54.3)	104 (59.1)	0.60
Male	16 (45.7)	72 (40.9)	
Comorbidity			
Present	17 (48.6)	85 (48.3)	0.97
Absent	18 (51.4)	91 (51.7)	
ECOG PS			
0	15 (42.9)	74 (42.0)	0.96
1	18 (51.4)	91 (51.7)	
2	2 (5.7)	11 (6.2)	
BMI			
<18.5, underweight	1 (3.1)	5 (3.8)	0.98
18.5–24.9, normal	17 (53.1)	73 (56.2)	
25–29.9, overweight	11 (34.4)	39 (30.0)	
≥30, obese	3 (9.4)	13 (10.0)	
Smoking	14 (40)	76 (43.2)	0.73
None	21 (60)	100 (56.8)	
Alcohol use	3 (8.6)	16 (9.1)	0.92
None	32 (91.4)	160 (90.9)	
Histological subtype			
Intestinal	7 (21.2)	37 (24.3)	0.70
Pancreaticobiliary/mix	26 (78.8)	115 (75.7)	
pT stage			
T1	3 (8.6)	21 (11.9)	0.66
T2	11 (31.4)	49 (27.8)	
T3	20 (57.1)	92 (52.3)	
T4	1 (2.9)	14 (8.0)	
pN stage			
N0	13 (38.2)	65 (37.1)	0.77
N1	16 (47.19)	91 (52.0)	
N2	5 (14.7)	19 (10.9)	
LNR			
0	14 (41.2)	61 (41.9)	0.84
<0.2	10 (29.4)	47 (29.4)	
0.2–0.4	5 (14.7)	30 (18.8)	
≥0.4	5 (14.7)	16 (10.0)	
THLN			
≥16	18 (52.9)	75 (48.1)	0.61
<16	16 (47.1)	81 (51.9)	
Surgical margin			
R0	33 (97.1)	160 (92.0)	0.29
R1	1 (2.9)	14 (8.0)	
Pancreatic invasion			
Present	21 (61.8)	98 (61.6)	0.98
Absent	13 (38.2)	61 (38.4)	
LVI			
Present	23 (67.6)	121 (70.8)	0.71
Absent	11 (32.4)	50 (29.2)	
PNI			
Present	19 (55.9)	87 (50.3)	0.55
Absent	15 (44.1)	86 (49.7)	
Biliary obstruction			
Present	16 (45.7)	82 (47.4)	0.86
Absent	19 854.3)	91 (52.6)	
CA 19-9 U/mL			
<150	30 (85.7)	129 (81.6)	0.57
≥150 (if bilirubin is high: 300)	5 (14.3)	29 (18.4)	
Albumi, g/dL			
<3.5	7 (20)	44 (27.3)	0.37
≥3.5	28 (80)	117 (72.7)	
Hemoglobin, g/dL			
<12 for women; 13 for men	15 (44.1)	83 (50.6)	0.49
≥3.5	19 (55.9)	81 (49.4)	
Neutrophils, ×10^9^/L			
≥7000	28 (84.8)	137 (82.0)	0.69
<7000	5 (15.2)	30 (18.0)	
Lymphocyte, ×10^9^/L			
<1500	8 (24.2)	57 (34.3)	0.26
≥1500	25 (75.8)	109 (65.7)	
Platelets, ×10^9^/L			
<500	34 (97.1)	156 (91.2)	0.23
≥500	1 (2.9)	15 (8.8)	
Radiotherapy			
Received	5 (14.3)	76 (43.2)	0.001
None	30 (85.7)	100 (56.8)	

LNR: lymph node ratio (positive lymph node/total harvested lymph nodes); THLN: total harvested lymph nodes; LVI: lymphovascular invasion; PNI: perineural invasion.

**Table 2:** Univariate and multivariate analyses of effect of clinicopathological and treatment-related parameters on disease-free survival.

	Univariate Analysis, Mo	*p* Value	Multivariate Analysis	*p* Value
	(95%CI)		HR (95%CI)	
Age, years				
(median; range)				
<65	31.0 (24.1–37.9)	0.58		
≥65	24.0 (8.8–39.2)			
Gender				
Female	31.0 (27.1–34.9)	0.56		
Male	23.0 (19.8–26.1)			
Comorbidity				
Present	24.0 (14.2–33.8)	0.90		
Absent	31.0 (22.5–39.4)			
ECOG PS				
0	33 (28.7–37.3)	0.088	1 (ref)	0.91
1	24 (15.1–32.9)		0.9 (0.6–1.5)	
2	11.0 (8.2–13.7)		1.1 (0.5–2.7)	
BMI				
<18.5, underweight	10.0 (3.0–24.0)	0.82		
18.5–24.9, normal	31.0 (22.1–39.9)			
25–29.9, overweight	24.0 (13.5–34.6)			
≥30, obese	21.0 (16.1–25.6)			
Smoking	27.0 (16.9–37.0)	0.72		
None	31.0 (22.6–39.1)			
Alcohol use	30 (23.9–36.0)	0.64		
None	14 (3.0–31.47)			
Histological subtype				
Pancreaticobiliary/mix	27.0 (20.8–33.1)	0.28		
Intestinal	34.0 (26.8–41.2)			
pT stage			-	
T1	34.0 (29.3–38.7)	0.059		
T2	37.0 (22.9–51.0)			
T3	23.0 (19.0–26.6)			
T4	10.0 (8.2–11.7)			
pN stage			-	
N0	43.0 (9.1–76.9)	<0.001		
N1	23.0 (18.4–27.6)			
N2	14.0 (4.8–23.2)			
Stage				
I (T1-2N0)	72 (Non-reached)		1 (ref)	
II (T3N0)	33.0 (28.3–37.7)	<0.001	1.1 (0.5–2.7)	0.26
IIIA (T 1-3N1)	24.0 (17.2–30.8)		1.5 (0.7–3.2)	
IIIB (T4 or N2)	13.0 (3.8–22.1)		2.0 (0.9–4.7)	
LNR				
0	43.0 (15.5–70.5)	0.007	-	
<0.2	21.0 (14.6–27.3)			
0.2–0.4	33.0 (7.7–58.2)			
≥0.4	17.0 (9.0–24.9)			
THLN				
≥16	24.0 (15.4–32.5)	0.49		
<16	30.0 (20.1–39.9)			
Surgical margin				
R0	32.0 (25.1–38.8)	0.001	2.1 (1.1–4.2)	0.035
R1	14.0 (6.4–21.6)			
Pancreatic invasion				
Present	19.0 (12.6–25.3)	<0.001	1.62 (0.91–2.8)	0.073
Absent	51.0 (14.6–87.4)			
LVI				
Present	23.0 (17.5–28.4)	<0.001	2.1 (1.2–3.8)	0.010
Absent	NR			
PNI				
Present	21.0 (16.6–25.4)	0.010	0.94 (0.58–1.50)	0.80
Absent	34.0 (22.6–45.4)			
Biliary obstruction				
Present	23.0 (13.9–32.1)	0.017		
Absent	33.0 (18.8–47.1)			
CA 19-9 U/mL				
<150	31.0 (23.9–38.0)	0.003	1.70 (0.9–3.1)	0.076
≥150 (if bilirubin is high: 300)	13.0 (8.3–17.7)			
Albumin, g/dL				
<3.5	24.0 (7.4–40.6)	0.20		
≥3.5	30.0 (24.4–35.5)			
Hemoglobin, g/dL				
<12 for women; 13 for men	24.0 (17.9–30.0)	0.15		
≥12–13	32.0 (15.4–48.6)			
Neutrophils, ×10^9^/L				
≥7000	24.0 (16.9–31.1)	0.15		
<7000	32.0 (22.9–41.0)			
Lymphocyte, ×10^9^/L				
<1500	27.0 (20.0–33.9)	0.68		
≥1500	31.0 (17.9–44.1)			
Platelets, ×10^9^/L				
<500	15.0 (2.0–31.6)	0.27		
≥500	30.0 (23.9–36.1)			
Radiotherapy				
Received	29.0 (21.5–36.5)	0.45		
None	31.0 (21.2–40.8)			
mFOLFIRINOX	NR	0.034	2.22 (1.1–4.6)	0.034
The other treatments	27.0 (20.7–33.3)			
mFOLFIRINOX	NR	0.064		
FU-doublet	16.0 (1.5–30.6)			
Gemcitabine doublet	24.0 (17.1–30.9)			
Gemcitabine or 5-FU	33.0 (19.2–46.7)			
CRT	34.0 (18.8–35.7)			

LNR: lymph node ratio (positive lymph node/total harvested lymph nodes); THLN: total harvested lymph nodes; LVI: lymphovascular invasion; PNI: perineural invasion.

**Table 3:** Univariate and multivariate analyses of effect of clinicopathological and treatment-related parameters on overall survival.

	Univariate Analysis, Mo	*p* Value	Multivariate Analysis	*p* Value
	(95%CI)		HR (95%CI)	
Age, years				
(median; range)				
<65	59.0 (41.0–76.9)	0.045	1.1 (0.63–1.85)	0.76
≥65	44.0 (28.7–59.3)			
Gender				
Female	51.0 (34.7–67.3)	0.98		
Male	55.0 (35.9–74.0)			
Comorbidity				
Present	44.0 (30.7–57.3)	0.007	1.57 (0.94–2.6)	0.084
Absent	70.0 (31.2–108.8)			
ECOG PS			-	
0	52.0 (24.9–79.1)	0.009		0.90
1	55.0 (36.0–73.9)			
2	11.0 (3.0–34.8)			
BMI				
<18.5, underweight	11.0 (3.0–33.2)	0.226		
18.5–24.9, normal	51.0 (39.6–62.4)			
25–29.9, overweight	90.0 (32.9–147.1)			
≥30, obese	42.0 (29.3–54.7)			
Smoking	53.0 (36.8–69.2)	0.93		
None	51.0 (34.3–67.7)			
Alcohol use	52 (38.7–65.3)	0.78		
None	32 (3.0–109)			
Histological subtype				
Pancreaticobiliary/mix	44.0 (34.0–53.9)	0.56		
Intestinal	63.0 (41.7–84.3)			
Stage			-	
I (T1-2N0)	92 (59.2–124.7)			
II (T3N0)	44.0 (10.0–77.8)	0.098		0.59
IIIA (T1-3N1)	48.0 (33.4–61.6)			
IIIB (T4 or N2)	44.0 (25.0–56.8)			
LNR				
0	67.0 (52.4–81.6)	0.42		
<0.2	44.0 (23.9–64.1)			
0.2–0.4	51.0 (43.9–58.1)			
≥0.4	36.0 (17.6–54.4)			
THLN				
≥16	51.0 (38.8–63.2)	0.51		
<16	52.0 (31.4–72.6)			
Surgical margin				
R0	55.0 (42.6–67.0)	0.008	1.87 (0.79–4.3)	0.15
R1	30.0 (27.7–32.3)			
Pancreatic invasion				
Present	36.0 (25.1–46.8)	<0.001	1.61 (0.90–2.86)	0.107
Absent	73.0 (46.1–99.9)			
LVI				
Present	44.0 (36.2–51.8)	0.020	1.33 (0.8–2.6)	0.23
Absent	92.0 (43.8–140.1)			
PNI				
Present	51.0 (37.1–64.9)	0.016	1.38 (0.82–2.34)	0.62
Absent	73.0 (42.3–103.6)			
Biliary obstruction				
Present	51.0 (37.6–64.4)	0.61		
Absent	63.0 (40.9–85.1)			
CA 19-9 U/mL				
<150	55.0 (41.4–68.6)	0.001	1.62 (0.83–3.2)	0.11
≥150 (if bilirubin is high: 300)	28.0 (20.8–35.1)			
Radiotherapy				
Received	52.0 (32.6–71.4)	0.607		
None	52.0 (40.3–63.7)			
mFOLFIRINOX	NR	0.071	3.24 (1.02–10.9)	0.046
The other treatments	51.0 (41.6–60.4)			

LNR: lymph node ratio (positive lymph node/total harvested lymph nodes); THLN: total harvested lymph nodes; LVI: lymphovascular invasion; PNI: perineural invasion.

**Table 4:** Treatment-related toxicities in patients receiving adjuvant therapy.

Adverse Event	FOLFIRINOX Grade 1–2 *n* (%)	FOLFIRINOX Grade 3–4 *n* (%)	Other Tx Grade 1–2 *n* (%)	Other Tx Grade 3–4 *n* (%)	*p*-Value
Neutropenia	11 (31.4)	7 (20.0)	65 (36.9)	25 (14.2)	0.013
Febrile neutropenia	-	5 (14.7)	-	6 (3.5)	0.008
Anemia	19 (55.9)	2 (5.9)	92 (55.5)	3 (1.8)	0.164
Thrombocytopenia	10 (29.4)	2 (5.8)	51 (31.3)	4 (2.4)	0.234
Fatigue	26 (75.8)	4 (10.3)	109(62.1)	4 (2.6)	0.043
Appetite loss	21 (60.7)	2 (7.1)	93 (54.3)	3 (2.0)	0.30
Diarrhea	11 (32.2)	0	27 (16.2)	0	0.034
Stomatitis	9 (25.9)	0	34 (20.2)	0	0.69
LFT abnormalities	12 (34.3)	0	36 (20.9)	2 (1.3)	0.37

## Data Availability

The datasets generated and/or analyzed during the current study are available from the corresponding author on reasonable request.
